# A missing dusk-side loss process in the terrestrial electron ring current

**DOI:** 10.1038/s41598-023-28093-2

**Published:** 2023-01-18

**Authors:** Bernhard Haas, Yuri Y. Shprits, Hayley J. Allison, Michael Wutzig, Dedong Wang

**Affiliations:** 1 GFZ German Research Centre for Geosciences, Potsdam, Germany; 2 Institute of Physics and Astronomy, University of Potsdam, Potsdam, Germany; 3 Department of Earth, Planetary, and Space Sciences, University of California, Los Angeles, CA USA

**Keywords:** Magnetospheric physics, Magnetospheric physics

## Abstract

The Earth’s magnetic field traps charged particles which are transported longitudinally around Earth, generating a near-circular current, known as the ring current. While the ring current has been measured on the ground and space for many decades, the enhancement of the ring current during geomagnetic storms is still not well understood, due to many processes contributing to its dynamics on different time scales. Here, we show that existing ring current models systematically overestimate electron flux observations of 10–50 keV on the nightside during storm onset. By analyzing electron drift trajectories, we show that this systematic overestimation of flux can be explained through a missing loss process which operates in the pre-midnight sector. Quantifying this loss reveals that the theoretical upper limit of loss has to be reached over a broad region of space in order to reproduce the observations. This missing loss may be attributed to inaccuracies in the parameterization of the loss due to chorus wave interactions, combined with the scattering by electrostatic electron cyclotron harmonic waves which is currently not included in ring current models.

## Introduction

One of the major characteristics of a geomagnetic storm is the enhancement of the particle flux in the terrestrial ring current. Due to the increased convection electric field and substorm injections during geomagnetically active periods, both electron and ion populations, consisting of trapped and quasi-trapped particles, are transported from the nightside geomagnetic tail structure inwards towards Earth. The enhancement of electron flux at energies of ~ 10’s keV in the ring current not only weakens the Earth’s net magnetic field, but may also form a hazardous environment for surface charging effects on spacecraft predominantly on the pre-midnight to dawn side, which may lead to satellite anomalies^1–3^. Ring current electrons interacting with plasma waves can be scattered to lower altitudes, where they collide with atmospheric molecules causing diffuse aurora^4^ and the formation of Nitrogen oxides (NO$_\text {x}$) in the lower thermosphere^5,6^. The produced NO$_\text {x}$ can then progress down to the stratosphere, where it causes the catalytic destruction of ozone. Due to the important influence of the ring current on geospace and the atmosphere, it is vital that we understand and are able to accurately simulate the dynamics of particles in this region.

The complex motion of charged particles in the Earth’s magnetic field can be simplified by describing the state of particles in terms of adiabatic invariants, variables that are usually conserved in the absence of plasma waves. The first and second adiabatic invariants describe the gyro and bounce motion of particles in the Earth’s magnetic field^7^, and their conservation leads to acceleration of particles as they are transported inwards towards Earth, into regions of stronger magnetic field. The energization process of thermal electrons up to 10–100 $\hbox {keV}$ provides energy to plasma waves which, in turn, resonate with radiation belt electrons, accelerating them up to multi-$\hbox {MeV}$^8,9^. Counteracting this source process, the precipitation of ring current electrons into the atmosphere due to scattering through wave–particle interactions is regarded as the main loss process for electrons^4^. The competition of source and loss processes leads to rapid particle flux changes during a geomagnetic storm with time scales of less than an hour. To model the flux enhancement during geomagnetic storms, kinetic models of ring current electrons have been developed. The Fokker–Planck equation, which has been proven to be a powerful tool for describing radiation belt dynamics^10,11^, is also capable of describing ring current electron dynamics in a convective–diffusive manner^12–14^.

**Figure 1:**
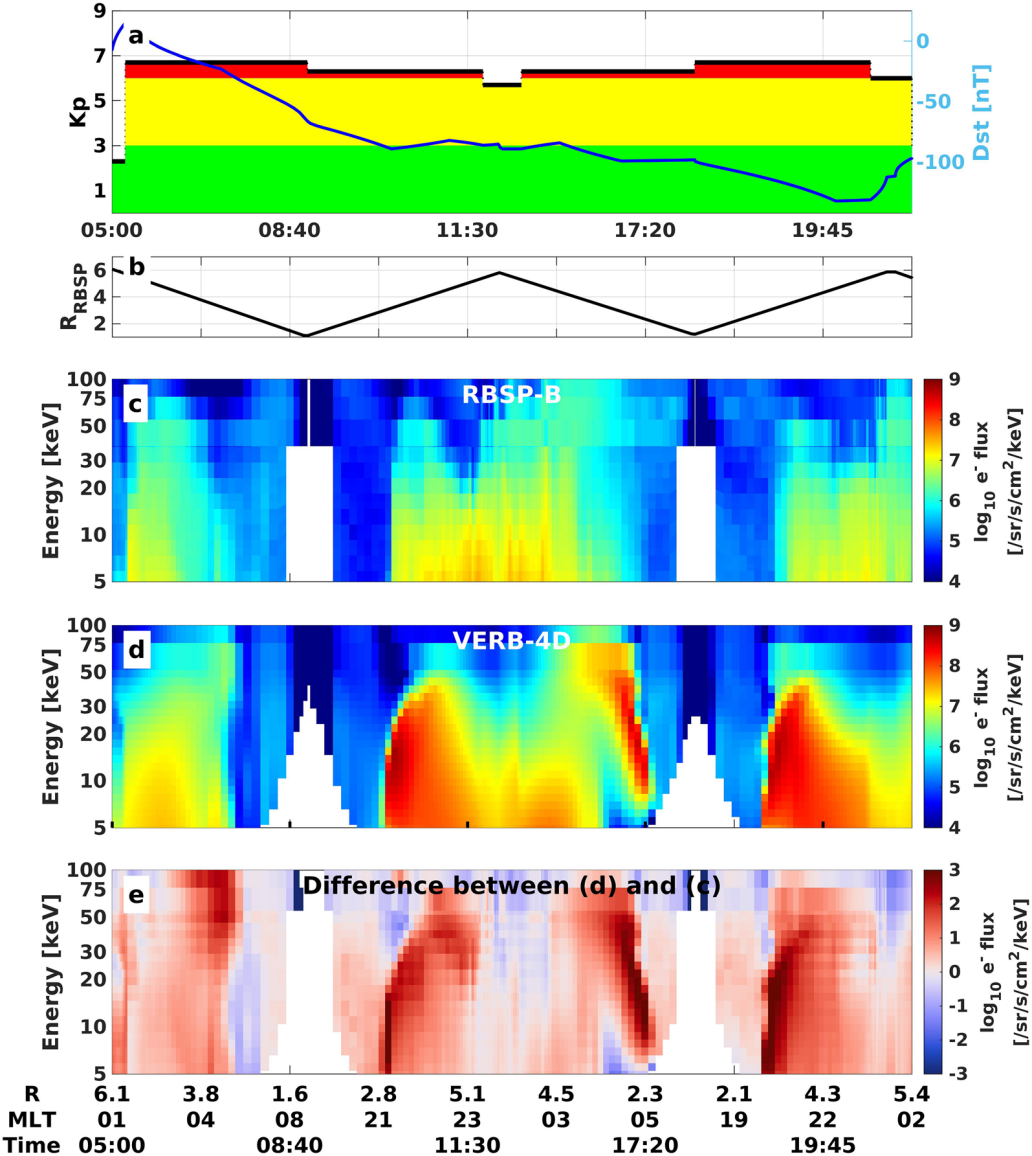
Flux spectrogram comparison during 17 March 2013. **(a)** Kp and Dst timeline. **(b)** Radial distance of the RBSP-B satellite mapped to the equatorial plane. Electron flux spectrogram at $50^\circ$ local pitch-angle **(c)** observed by the RBSP-B satellite and **(d)** predicted by VERB-4D. **(e)** Logarithmic difference between simulation results and observations.

Electrons first enter the inner magnetosphere on the nightside and drift eastward around the Earth towards the dayside. Validating the initial nightside enhancement is therefore key to further investigate the electron dynamics on the dawn and dayside. Simulations of the first few hours of intense geomagnetic storms overestimate satellite observations of the ${10}{-}{50}\,{\hbox {keV}}$ electron flux close to Earth on the nightside (see Fig. 1). This phenomenon is not specific to a given storm event but rather appears as a systematic difference between simulations and reality at the start of geomagnetic storms^15^.

Here we report that this overestimation during storm onset is unlikely caused by inaccuracies of the source terms, but rather due to not strong enough loss of electrons. We find that increased loss in the pre-midnight sector is necessary to reproduce observations, which reaches the physical theoretical upper limit of loss over a broad region, the strong diffusion limit. We attribute this missing loss to inaccuracies in the electron loss associated with chorus wave scattering, combined with scattering by electrostatic electron cyclotron harmonic (ECH) waves, which is currently not included in modern ring current models.

## Results

### Simulation of the March 2013 St. Patrick’s Day storm

To investigate the loss and source processes, we focus on the geomagnetic storm occurring on 17 March 2013, because of its clear distinction between low and high geomagnetic activity. This event is considered as a strong storm event within the Van Allen Probes’ era, and is also known as the St. Patrick’s Day event, examined in several works^16–19^. As shown in Fig. 1a, the storm started at 05:00 UTC and the Dst index (a measure of the changes in the Earth’s magnetic field strength in the near equatorial region) first increased in response to the day-side compression of the magnetosphere. At 06:00 UTC, Dst began to decline signifying the commencement of the main phase of the storm, which lasted for ~ 14 h. Upon storm onset, the Kp index (a semi-logarithmic scale of the geomagnetic field disturbance) increased sharply to 7- and remained between 6- and 7- throughout the storm.

Figure 1c shows the electron flux observations measured by the Helium, Oxygen, Proton, and Electron (HOPE)^20^ and the Magnetic Electron Ion Spectrometer (MagEIS)^21^ instruments mounted on the Radiation Belt Storm Probes (RBSP) B satellite, which has its apogee at 00-01 magnetic local time (MLT) during this event. For the sake of clarity, data is plotted on a modified time axis, such that the radial distance of the satellite changes linearly (Fig. 1b), clearly displaying the region closer to Earth, which is only briefly traversed by the satellite^15^. Observing the incoming particles on the nightside during the storm, the satellite measured enhanced electron flux down to radial distances of ~ 3 Earth radii ($R_E$). The sharp drop of flux at 3 $R_E$ appositely coincides with the Alfvén layer, the layer separating closed and open orbits for zero energy particles, which are not able to penetrate deeper under these geomagnetic conditions, assuming static electric and magnetic fields.

We test if we can reproduce the enhancement with the Versatile Electron Radiation Belt (VERB-4D) model, which solves for advection and diffusion of particles in the four dimensions of magnetic local time (MLT), radial distance from Earth, and first and second adiabatic environments. In this work, we are using empirically derived models parameterized by the Kp index to describe the electric field, magnetic field, and flux at the outer boundary condition. Electron loss is incorporated by means of an exponential loss term, associated with chorus^22,23^ and hiss^24^ scattering. The full model setup is described in the “Methods” section.

**Figure 2:**
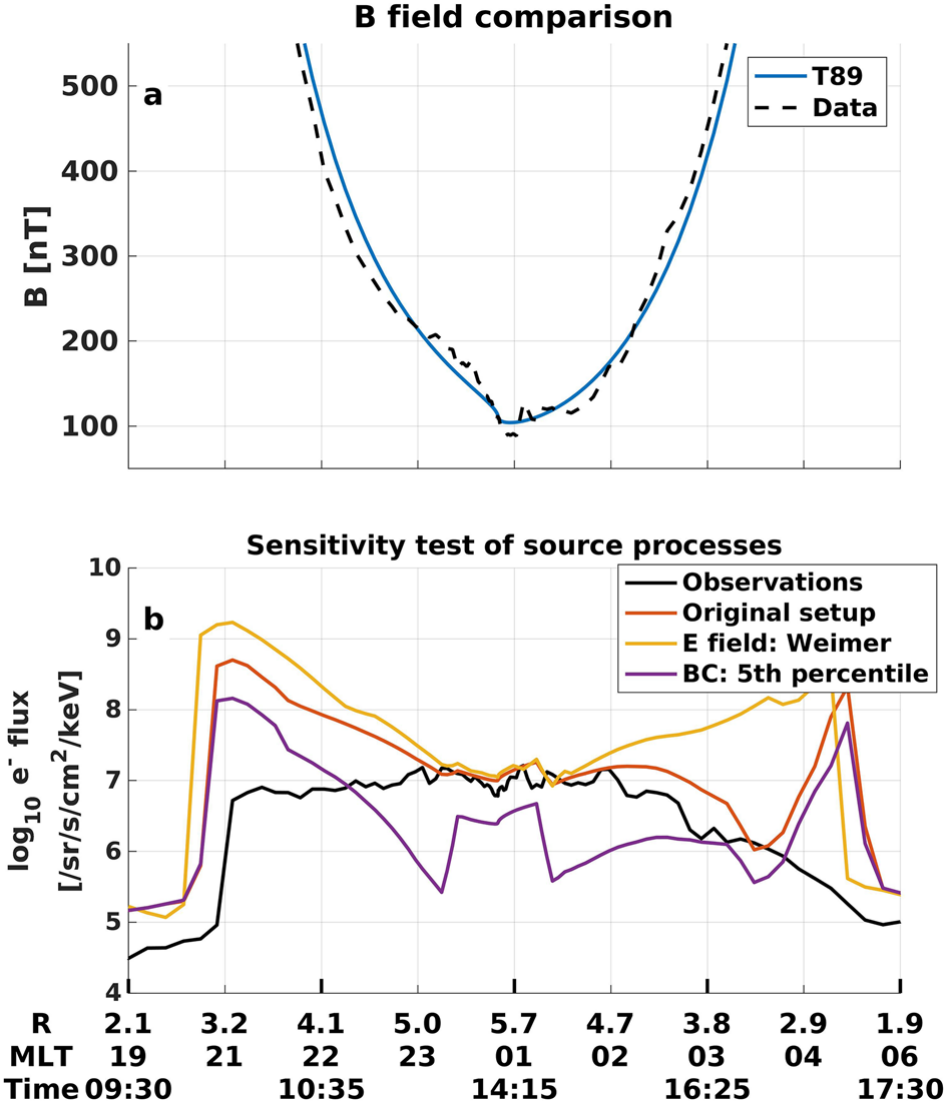
Sensitivity test of source terms of VERB-4D for the second orbit of RBSP-B during the March 2013 storm. **(a)** Comparison between T89 and RBSP EMFISIS observations of magnetic field magnitude along the RBSP-B orbit. **(b)** VERB-4D simulation results for $10\,\hbox {keV}$ electrons using the original setup (see Fig. 1), the Weimer electric field model^61^, and statistically low flux values at the outer radial boundary given by the 5th percentile of electron flux predicted by the Denton model^52^.

Comparing the simulation results with RBSP-B observations along the satellite’s orbit (Fig. 1d), VERB-4D overestimates the electron flux consistently between 5 and ${50}\,{\hbox {keV}}$ for three consecutive trajectories of the satellite at radial distances between 2.5 and 5 $R_E$. The overestimation has a maximum around the Alfvén layer at energies between 10 and ${20}\,{\hbox {keV}}$, while electrons at energies $> 60\,{\hbox {keV}}$ are reproduced relatively well. An imbalance of the source and loss processes of the model can be caused by inaccuracies of the outer radial boundary conditions, the ambient electric and magnetic field (used for the drift velocity calculation), or the assumed electron lifetimes. A comparison of the empirical magnetic field model used in this work against observations of the RBSP-B Electric and Magnetic Field Instrument Suite and Integrated Science (EMFISIS) instrument^25^ shows good agreement throughout the storm’s main phase (see Fig. 2a, Supplementary Fig. S1), making it an unlikely candidate for causing the large overestimation. To test whether modelling errors of the electric field or boundary conditions are causing the overestimation, additional simulations are performed using a different electric field model, and statistically unlikely low flux levels at the model’s outer radial boundary (see “Methods” section). As seen in Fig. 2b, this does not resolve the observed discrepancies between model and reality, as the overestimation still persists. Our sensitivity test indicates that the discrepancies between the model and observations in Fig. 1 cannot be explained by inaccuracies of the source processes alone, but additional loss has to be applied in order to reproduce flux observations.

### Localization and quantitative description of missing loss process

Since measurements for this event are taken on the nightside, particles travel only a short distance within geosynchronous orbit (GEO) before they are measured by the satellite. By investigating the particles’ drift trajectories, one can find their origin at GEO and their drift path through the inner magnetosphere.

**Figure 3:**
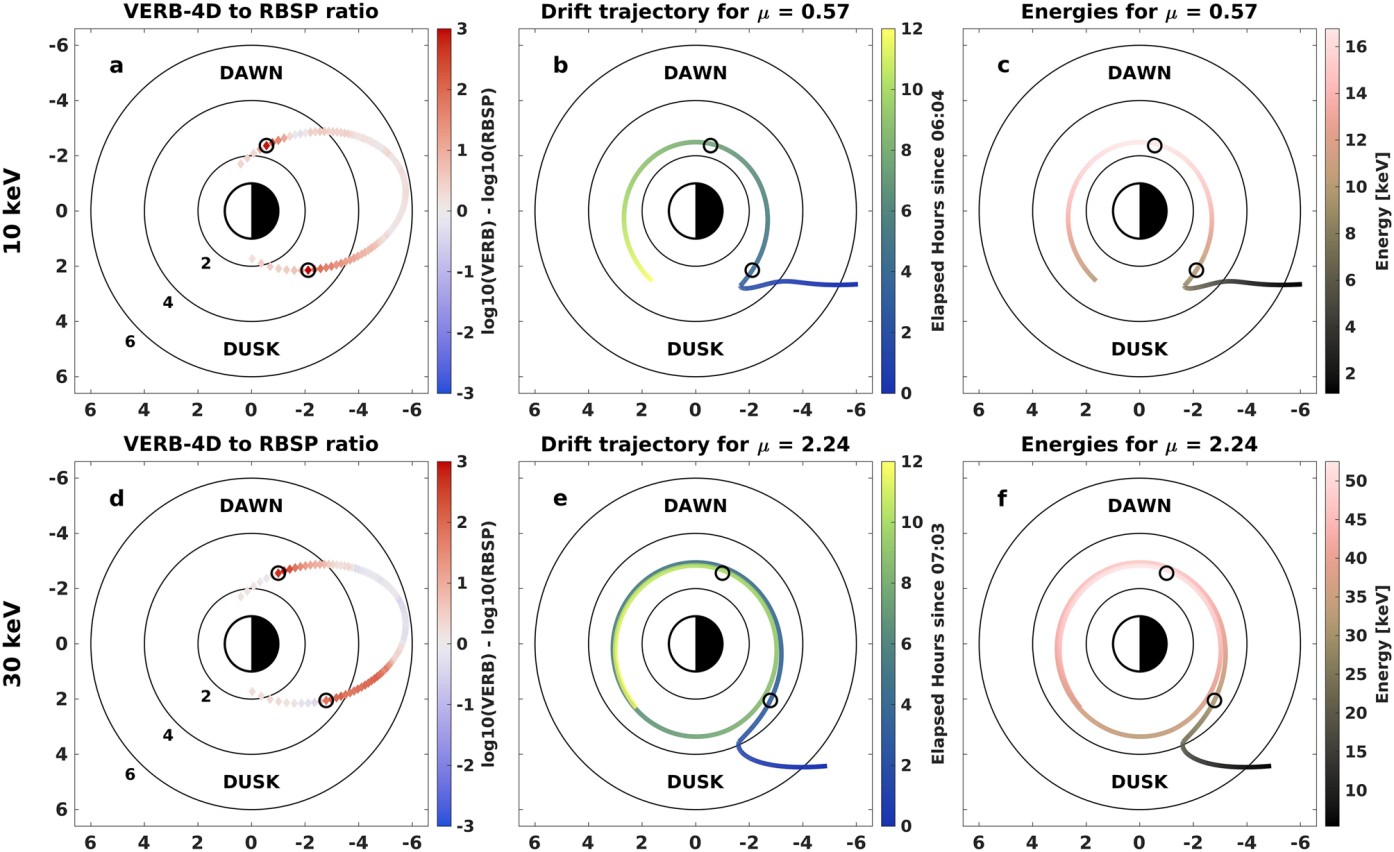
Spatial location of overestimation and the electrons’ drift paths. **(a)** Comparison between flux measured by RBSP-B and VERB-4D for the orbit starting around 09:00 and $10\,\hbox {keV}$. The black circle indicates the innermost point, where overestimation was greater than one order of magnitude on the given trajectory. **(b)** Calculated drift trajectory for a $10\,\hbox {keV}$ and $50^\circ$ pitch-angle electron crossing the point of innermost overestimation on the dusk-side indicated again by the black circle. Color-coded is the elapsed time of the drift starting at the injection of the particle at GEO. **(c)** Same drift paths as in wave-particle interactions but now color-coded is the energy of particles along the drift path. **(d–f)** Same format as **(a–c)** but for $30\,\hbox {keV}$ particles.

Firstly, we find the innermost location where the overestimation was greater than one order of magnitude along the RBSP-B dusk-side trajectory, starting around 09:00 UTC, for 10 and ${30}\,{\hbox {keV}}$ electrons (dusk-side circle in Fig. 3a,d). The drift trajectory of particles forming this overestimation is calculated by tracing them forwards and backwards in time while conserving their first and second adiabatic invariants. The resulting drift trajectories have their origin at GEO in the pre-midnight sector (see Fig. 3b,e). From there, particles are transported directly to lower radial distances due to the strong sunward convection, before starting their drift around Earth. Both particle populations are travelling away from GEO for around 4 hours before they are measured by the satellite. Figure 3c,f display the change in energy along the particles’ trajectories because of adiabatic acceleration due to conservation of the first adiabatic invariant. Electrons at ${1}\,{\hbox {keV}}$ entering the simulation domain at GEO will end up forming the ${10}\,{\hbox {keV}}$ overestimation, while the ${30}\,{\hbox {keV}}$ overestimation is formed by particles with energies of ${5}\,{\hbox {keV}}$ at GEO.

Focusing now on the innermost location where the overestimation was greater than one order of magnitude along the subsequent pass of the satellite, which occurred on the dawn-side (dawn-side circle in Fig. 3), one can see that the dawn-side overestimation is associated with the same electron trajectories as the dusk-side flux overestimation. This demonstrates that both errors potentially stem from the same missing loss process, happening before the particles are measured by the satellite on the dusk-side. As a result, to resolve the overestimation on the dusk-side, the loss process must be located in the pre-midnight sector and affect electrons with energies between 1 and ${30}\,{\hbox {keV}}$. Furthermore, as the flux is overestimated by VERB-4D across radial distances of 3–5 $R_E$, the loss process must also act over these distances.

To quantify the missing loss process, we construct empirical electron lifetimes applied to the pre-midnight sector, so that the VERB-4D results would approximately match the RBSP-B observations. For simplicity, we assume the same spatial and Kp dependence as the chorus lifetimes, assume a linear dependence in energy, and fit the parameters by consecutive iterations (see “Methods” section). We find that with the addition of empirical lifetimes, the strong diffusion limit^26^ (a theoretical limit of electron loss rate due to finite lifetimes within the loss cone) is reached in the whole pre-midnight sector at large radial distances, indicating very strong loss (see Supplementary Fig. S2). Close to dusk, the missing loss process has to act down to radial distances of $\sim 3 R_E$, while closer to midnight, it is sufficient to add the empirical loss above radial distances of $\sim 4 R_E$. Because of the strongly eroded plamasphere during this storm, these radial distances reside in the low density plasmatrough.

**Figure 4:**
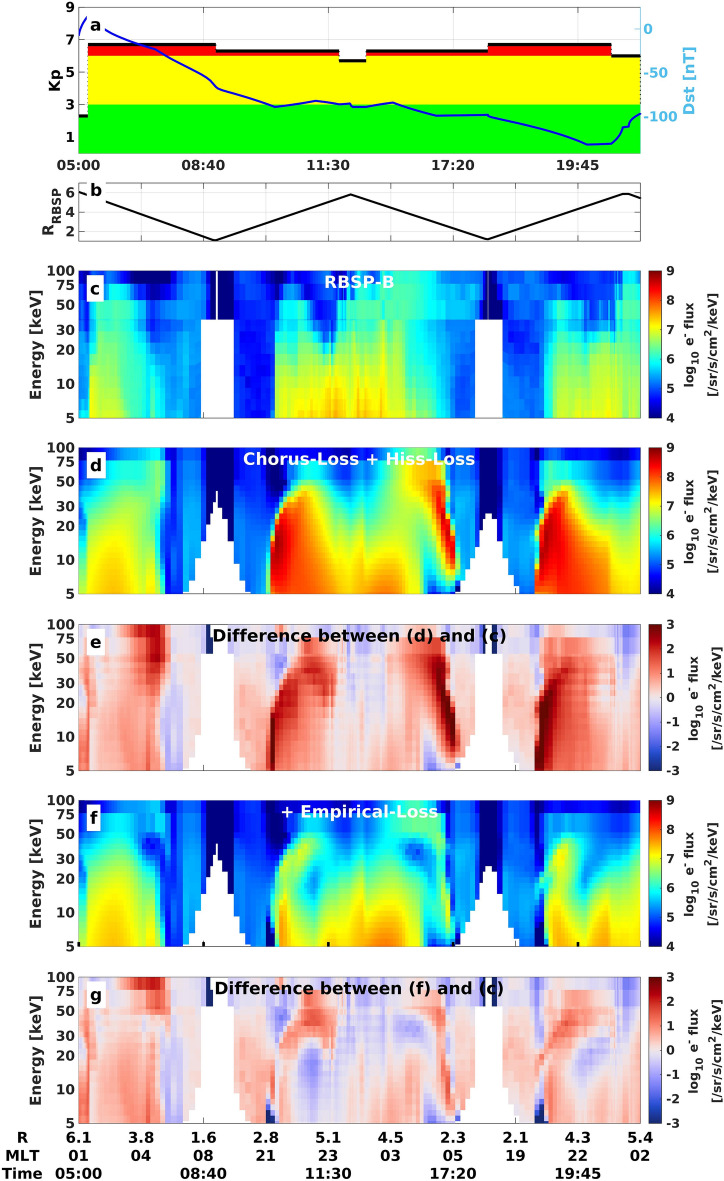
VERB-4D simulation using the derived empirical lifetimes. **(a)** Kp and Dst timeline. **(b)** Radial distance of the RBSP-B satellite mapped to the equatorial plane. Electron flux spectrogram at $50^\circ$ local pitch-angle **(c)** observed by the RBSP-B satellite, **(d)** predicted by VERB-4D using chorus and hiss lifetimes, and **(f)** predicted by VERB-4D adding the empirical lifetimes. Logarithmic difference between observations and **(e)** the simulation without empirical lifetimes, **(g)** with empirical lifetimes.

When we apply the additional empirical lifetimes to the VERB-4D simulations, particles are scattered by the strong loss in regions of strong diffusion and only a fraction are able to reach very low radial distances, in agreement with the observations (see Fig. 4). Therefore, the overestimation close to Earth is strongly decreased or eliminated entirely for all considered energies on both the dusk and the dawn-side, showing that a missing loss in the pre-midnight sector is indeed mainly responsible for the overestimation on the dawn-side.

## Discussion

We identified a systematic discrepancy between model predictions and observations during intense storm times in the inner magnetosphere. We have shown that inaccuracies of the source processes are unlikely to be responsible for these overestimations, by validating the magnetic field model against satellite observations and performing additional simulations using two different electric field models and lower flux at the outer radial boundary. Theoretically, it could be possible that both empirical electric field models overestimate the electric field strength during the storm, leading to an overestimation of particle flux. However, it has been shown that empirical models tend to underestimate the electric field strength during this particular event^27^. Furthermore, ring current simulations including self-consistent modelling of electric and magnetic fields show similar signatures of overestimation of low energy electron flux during this particular storm^28,29^. Nevertheless, due to the great variability of the electric field during storms^30^, the localization and quantification of the empirical loss should be treated with caution.

By looking at the drift trajectories of incoming particles, we were able to locate a missing loss process, which is capable of resolving the overestimations of our simulations. Although it is not possible to exactly locate the missing loss using this method, we found that the loss must reside in the pre-midnight sector in order to affect the overestimations on both the dusk and dawn-side. In order to quantify the loss to some extent, we fitted the VERB-4D simulation to reproduce the satellite observations by assuming a certain region of additional loss and fitted the parameters through trial and error. While the additional loss on the dusk-side resolved the overestimation at radial distances of $\sim 3 R_E$, it may be slightly too strong at higher radial distances, as regions which were overestimated moderately in the original simulation are now underestimated (see Fig. 4 Panel g, around 11:00). This indicates that the empirically constructed lifetimes probably do not hold the same spatial dependence as the lifetimes associated with chorus wave scattering and a more complicated fitting procedure would be necessary to perfectly fit the observations. Furthermore, the fitting solution is not unique and inaccuracies in the boundary conditions, electric field and the other loss models also may influence the prediction heavily. Therefore, the empirical lifetimes constructed in this work should be treated as only providing an indication as to where the missing loss process may operate and how strong it is. Nevertheless, we showed, that the total loss has to be extremely strong, reaching the theoretical upper limit of strong diffusion in our case over a broad region at large radial distances from Earth.

The main physical mechanism scattering electrons into the atmosphere and therefore producing loss from the magnetic trapping region are wave-particle interactions. Therefore, the question arises which wave population is responsible for the missing loss process observed in this work. One possible candidate are chorus waves. Although loss by chorus waves is already included in modern electron ring current models^12,13,29^, it is difficult to accurately estimate the impact of chorus waves on the electron population. Statistical models of the wave properties^22,31^ and cold plasma density are used to derive diffusion coefficients and electron lifetimes. These models may not be accurate during strong storm events, since statistics for such events are poor and models do not capture the characteristics^32,33^. In particular, the effect of cold plasma density variability on the scattering rates of electrons has been proven to be very significant^8,34^, while static models of the cold plasma environment completely misrepresent the density during strong storm events^8^. A comparison between the cold plasma density derived from EMFISIS measurements^35^ and the Sheeley model^32^ for the March 2013 storm reveals that the Sheeley model overestimates the density inside the plamsatrough for most of the storm (see Supplementary Fig. S3). Due to the uncertainties in wave properties and cold plasma density, it is not surprising that studies of event specific diffusion coefficients have shown great deviations from statistically derived coefficients^36,37^, including the coefficients of energetic electrons at dusk^28^.

Other types of waves may be also responsible for the missing loss process. ECH waves are currently not included in VERB-4D, although they are capable of very efficiently scattering electrons of a few eV up to ${10}\,{\hbox {keV}}$^38–40^. Statistical studies of satellite measurements show that ECH waves occur mostly at radial distances of 5 $R_E$ and above on the nightside, including the pre-midnight sector^39,41,42^, where the missing loss process operates. Therefore, ECH waves are very capable to scatter ${1}\,{\hbox {keV}}$ electrons at GEO, diminishing the overestimation of ${10}\,{\hbox {keV}}$ electrons tremendously at radial distances of $\sim 3 R_E$.

To determine whether ECH waves or chorus waves are responsible for the missing loss process, more sophisticated wave models of these types of waves have to be developed. It is particularly important to describe the wave properties during strong storms more accurately. As the dispersion relation of plasma waves and therefore the scattering of electrons strongly depends on the surrounding cold plasma density, simulations including dynamical cold plasma density models^43,44^ need to be performed to correctly model wave-particle interactions.

## Methods

### VERB-4D model setup

The four-dimensional Versatile Electron Radiation Belt code (VERB-4D)^45^, used to simulate radiation belt^45^, plasmasphere^44^ and ring current dynamics^14^, solves the diffusion–convection equation in MLT, radial distance *R* and the two modified adiabatic invariants *V* and *K*^46^:

1 $$\begin{aligned} K=\frac{J}{\sqrt{8m_0\mu }}\,\,\text {and}\,\,V=\mu (K+0.5)^2, \end{aligned}$$

where $$\mu$$ and *J* are the first and second adiabatic invariants^7^ and $$m_0$$ is the electron rest mass. While the code is capable of solving a full diffusion setup, which includes diffusion in $$L^*$$, energy and pitch-angle, the pitch-angle diffusion may be incorporated into a separate loss term under the assumption that the pitch-angle distribution decays uniformly with the lowest normal mode^47,48^. Energy diffusion is not considered as it acting on larger time scales and is expected to not have a significant impact at the considered energies^49^. Radial diffusion is implemented using precomputed diffusion coefficients^50^, which are adapted to smoothly vanish below $${40}\,{\hbox {keV}}$$, as particles with longer drift times cannot be treated as diffusing in $$L^*$$^51^.

In this setup the following equation is solved in each time step:

2 $$\begin{aligned} \frac{\partial f}{\partial t}=\langle v_\varphi \rangle \frac{\partial f}{\partial \varphi } + \langle v_R \rangle \frac{\partial f}{\partial R} + \frac{1}{G} \frac{\partial }{\partial L^*} G \langle D_{L^*L^*} \rangle \frac{\partial f}{\partial L^*} - \frac{f}{\tau _{wave}}, \end{aligned}$$

where *f* denotes phase space density, *G* is a Jacobian, $\langle D_{L^*L^*} \rangle$ are the bounce averaged diffusion coefficients in $L^*$, $\langle v_\varphi \rangle$ and $\langle v_R \rangle$ are bounce averaged drift velocities in MLT and R, and $\tau _{wave}$ are lifetimes of electrons associated with wave–particle interactions.

The numerical grid is set up using 113 points in R, covering the range from 1 to 6.6 $R_E$, while the MLT dimension is divided into 97 grid points. We are using more spatial points compared to previous studies^14,15^ such that the region around the Alfvén layer is accurately resolved while keeping numerical diffusion across it at a minimum. The *V* and *K* dimensions are set up using 30 and 31 grid points, while making sure that the desired energy and pitch-angle range is defined for $R > 2$. The upper radial boundary is described by the statistical mean given by the Kp-dependent Denton model^52^, while the drift velocities are calculated using the Volland–Stern electric field^53,54^ with the Maynard–Chen Kp parameterization^55^ and the addition of a Kp-dependent subauroral polarization stream module^56^, and the T89 magnetic field model^57^. VERB-4D uses a 9th order numerical scheme^58–60^, which allows for the accurate estimation of the advective transport of electrons. The sensitivity test of the source processes is performed using the Weimer^61^ model of ionospheric electric potential mapped to the equatorial plane^14^, while the statistically low outer radial boundary flux values at GEO are described by the 5th percentile of the Denton model^52^.

The loss of electrons due to wave-particle interactions is described by the lifetimes associated with chorus scattering^23^ outside the plasmasphere and with hiss scattering^24^ inside the plasmasphere. We are using the parameterized chorus lifetimes derived from the diffusion coefficients at the edge of the loss cone. The plasmapause location is calculated using the model of Carpenter and Anderson^62^. The dayside magnetosphere introduces loss by magnetopause shadowing for high energy particles^63^. As we are only interested in the nightside flux of low energy electrons, the magnetopause location will not affect our simulation and is therefore not modelled.

The initial condition for the simulation is constructed using the last full pass of the RBSP-B satellite before the simulations begins while assuming MLT-isotropy.

### Calculation of drift trajectories

To calculate the drift trajectory of a single particle population with given energy, pitch-angle, start point, and start time, we first calculate $\mu$ and *K* of the particles, assuming a dipole magnetic field. By tracing the particles backwards in time, their origin at GEO can be identified, while the rest of their trajectory is found by tracing them forwards in time. Both tracing directions are implemented as forward Euler steps as described by the following equations:

3 $$\begin{aligned}&\text {Backward tracing:}&x^{n-1}&= x^{n} - \langle v \rangle (x^{n},\mu ,K,\text {Kp}) \cdot \Delta t, \nonumber \\&\text {Forward tracing:}&x^{n+1}&= x^{n} + \langle v \rangle (x^{n},\mu ,K,\text {Kp}) \cdot \Delta t, \end{aligned}$$

where the bounce averaged particle’s velocity $\langle v \rangle$ consists of the ExB drift and gradient-curvature drift of particles and depends on the particles’ position *x*, $\mu$, *K* and the current Kp value. We have used a very small time step of $\Delta t = {3}\,\hbox {s}$ to ensure stability of the explicit method.

### Construction of empirical lifetimes

Lifetimes representing the missing loss process are constructed by multiplying the lifetimes associated with chorus wave scattering by a derived factor, which is linearly dependent on energy. In this way, the empirical lifetimes inherit the Kp, R and MLT dependence, while the energy dependence can be tuned to optimize the simulation results. Assuming the empirical lifetime factor has the form of Eq. (4) in log space, where *E* is the kinetic energy of a particle, the problem of finding the correct parameters a and b is simple enough to be found by manually tuning the parameters and comparing simulation results to observations in an iterative manner.

4 $$\begin{aligned} \text {log10}(\tau _{\text {empirical}}) = \text {log10}(\tau _{\text {chorus}}) - \frac{a-E}{b}. \end{aligned}$$

We restrict the application of empirical lifetimes in space, energy and also only apply them if Kp is over a certain threshold.

As simulation results agree reasonably well for energies $\ge$ ${50}\,\hbox {keV}$ (see Fig. 1), we conclude that no additional loss is required above ${50}\,\hbox {keV}$. The missing loss process is therefore only active at low energy. On the dusk side (17–21 MLT), we have applied the empirical loss according to Eq. (4) down to R = 3, while we have applied such loss only for R $> 4.5$ closer to midnight (21–24 MLT), as higher loss at smaller radial distances led to underestimation of the observed electron flux. Regarding the Kp threshold, the St. Patrick’s Day event has a clear distinction between quiet time and storm onset, leading to the choice of applying the empirical lifetimes when Kp is greater than 5, resulting in applying them during the full storm period. Parameter *a* is set to ${50}\,\hbox {keV}$ to smooth out the lifetimes at high energies, while parameter *b* is determined by trial and error while comparing the simulation results to the RBSP observations, to be ${0.0312}\,\hbox {keV}$.

When the scattering rate of electrons is faster than the loss rate of particles inside the loss cone, the loss cone fills and eventually reaches a flat pitch-angle distribution. Because of this phenomenon, a theoretical lower limit of electron lifetime exists, also called the strong diffusion limit^26,64^. As a consequence, if the empirical lifetimes would suggest a lifetime below the strong diffusion limit^64^, the limit is used instead.

## Supplementary Information

Supplementary Figures.

## Data Availability

All RBSP-ECT data are publicly available at the website https://rbsp-ect.newmexicoconsortium.org/data_pub/rbspb/hope/level3/pitchangle/ and the RBSP-EMFISIS data at the website https://emfisis.physics.uiowa.edu/Flight/. Dst and Kp values are from the NASA OMNIWeb data explorer, accessible at https://omniweb.gsfc.nasa.gov/form/dx1.html.
