# Supplementary material for: A missing dusk-side loss process in the terrestrial electron ring current
Source: Sci Rep. 2023 Jan 18;13:970. doi: 10.1038/s41598-023-28093-2 (PMC9849215; doi:10.1038/s41598-023-28093-2)
Supplement: Supplementary file 1 — Supplementary Figures. [file 41598_2023_28093_MOESM1_ESM.pdf]

Supplementary Material for Article titled:

'A Missing Dusk-side Loss Process in the Terrestrial Electron Ring Current'

Bernhard Haas[1,2,*], Yuri Y. Shprits[1,2,3], Hayley J. Allison[1],

Michael Wutzig[1], and Dedong Wang[1]

1 GFZ German Research Centre for Geosciences, Potsdam, Germany
2 Institute of Physics and Astronomy, University of Potsdam, Potsdam, Germany
3 Department of Earth, Planetary, and Space Sciences, University of California, Los Angeles, CA, USA
* bhaas@gfz-potsdam.de

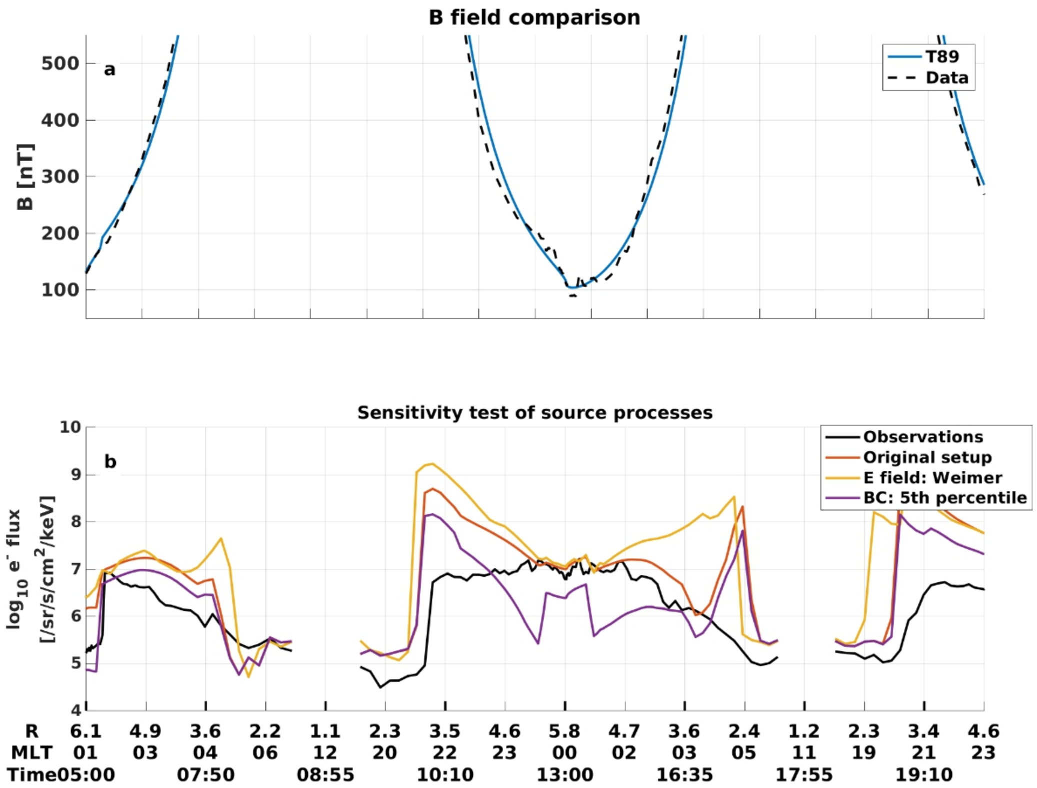

Figure S1. Sensitivity test of source terms of VERB-4D during the main phase of the March 2013 storm. **(a)** Comparison between T89 and RBSP EMFISIS observations of magnetic field magnitude along the RBSP-B orbit. **(b)** VERB-4D simulation results for 10 keV electrons using the original setup (see Figure 1), the Weimer electric field model, and statistically low flux values at the outer radial boundary given by the 5th percentile of electron flux predicted by the Denton model.

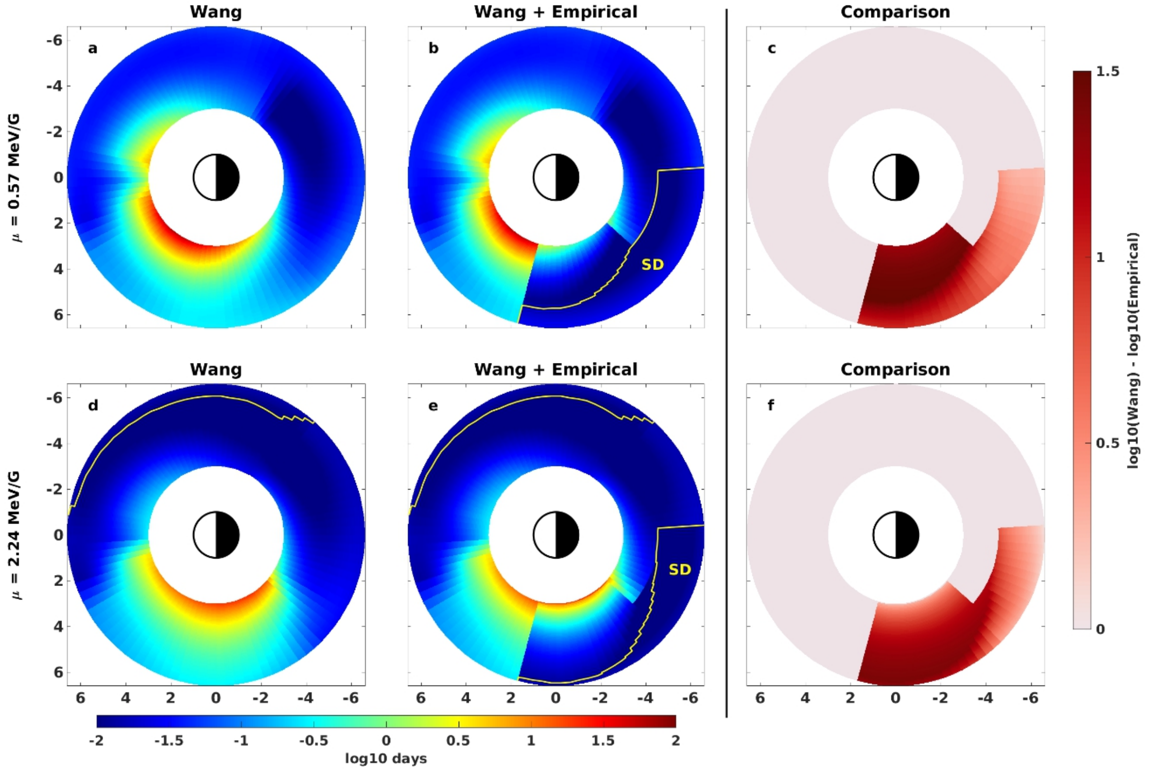

Figure S2. Comparison of empirical and chorus lifetimes. **(a)** Chorus lifetimes for μ = 0.57 $MeVG^{-1}$ **(b)** Chorus + empirical lifetimes derived in this work. **(c)** Comparison between the two in log10 space. Panels **(d to f)** have the same format but for μ = 2.24 $MeVG^{-1}$.

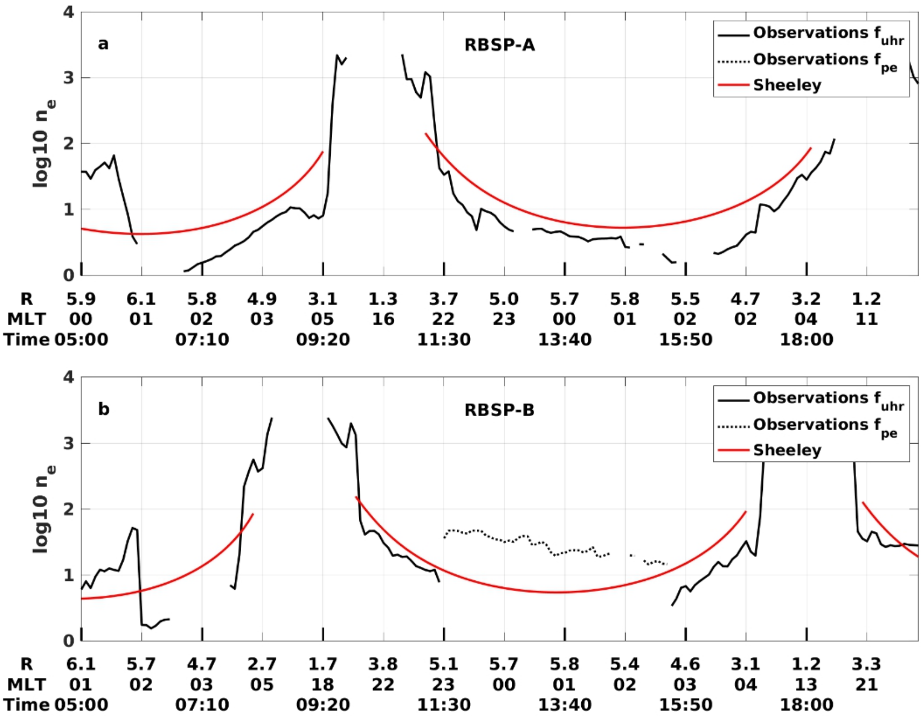

Figure S3. Comparison of cold plasma density observations measured by the RBSP satellites with the empirical Sheeley model **(a)** along the RBSP-A orbit, **(b)** along the RBSP-B orbit. The solid lines represent the density values calculated by identifying the upper hybrid frequency in the spectrogram, while the dotted lines represent measurements, where the upper hybrid resonance frequency could not be determined, and an upper limit of cold plasma density was calculated by identifying the low-frequency extend as a proxy for the electron plasma frequency. The plume-like structure in the plasmatrough seen in the RBSP-B measurements during the main phase of the storm are most likely artificial and not real.
